# Rapid and Significant Reduction in Size of Pituitary Adenoma in Children Treated with Fractionated Stereotactic Radiation Therapy: A Case Report

**DOI:** 10.1155/2011/187839

**Published:** 2011-09-29

**Authors:** Putipun Puataweepong, Mantana Dhanachai

**Affiliations:** Department of Radiology, Faculty of Medicine, Ramathibodi Hospital, Mahidol University, Bangkok 10400, Thailand

## Abstract

Radiation treatment in the management of pituitary adenoma in children is still a controversy due to the great concern of late radiation effect. Stereotactic radiation therapy is the modern radiation technique that is more widely applicable in pituitary adenoma because of the ability to give highly conformal radiation to the target organ and to reduce the radiation dose to normal tissue. A 15-year-old boy presented with postradiation therapy recurrence pituitary macroadenoma. His vision was useful only on the left eye. MRI revealed the recurrent macroadenoma with severe invasion of optic apparatus. Since no any other treatment option was to be given to this patient, we decided to reirradiate the patient with conventional fractionated stereotactic radiation. At 3 months of followup, MRI showed that the tumor had an early and significant reduction in size without any sign of toxicity.

## 1. Introduction

Pituitary adenomas are rare in children. They may be quite large, with extrasellar extension, and appear to be more invasive than those seen in adults [1–3]. Visual loss, when present, may be more severe and more likely to be associated with optic atrophy [3]. Transphenoidal surgery appears to be feasible and safe in children [1, 2]. Radiation therapy is indicated if surgical resection is not possible or recurrent or if hormone levels remain elevated following surgery. Late radiation complications such as panhypopituitarism, optic neuropathy, or radiation-induced malignancy are of the great concern for radiation in children. Stereotactic radiation (SRT) is the modern conformal radiation technique that is able to give highly conformal radiation to the target organ and reduce the radiation dose to normal tissue. Minimizing the radiation dose to normal brain of SRT may reduce the risk of developing late radiation-induced complication. In recent years, SRT is widely applicable in pituitary adenoma, especially in the case of high risk for late radiation complication such as reirradiated in recurrent cases or in the children. This communication presented a report case of the recurrent pituitary adenoma in children reirradiated with stereotactic radiation technique. 

## 2. Case Report

A 15-year-old boy was referred to our hospital (Ramathibodi Hospital, Bangkok, Thailand) because of recurrent pituitary macroadenoma.

In 2007, he presented with decreasing of right eye vision. MRI revealed pituitary macroadenoma. He had been transphenoidal with partial tumor removal followed by conventional external beam radiation therapy with the total dose of 5040 cGy in 28 fractions due to residual tumor. After surgery and radiation, he developed panhypopituitarism and was treated with eltroxin (100 mcg) 1.5 tab oral OD, prednisolone(5) 0.5 tab oral OD, and DDAVP 0.1 mL bid.

**Figure 1:**
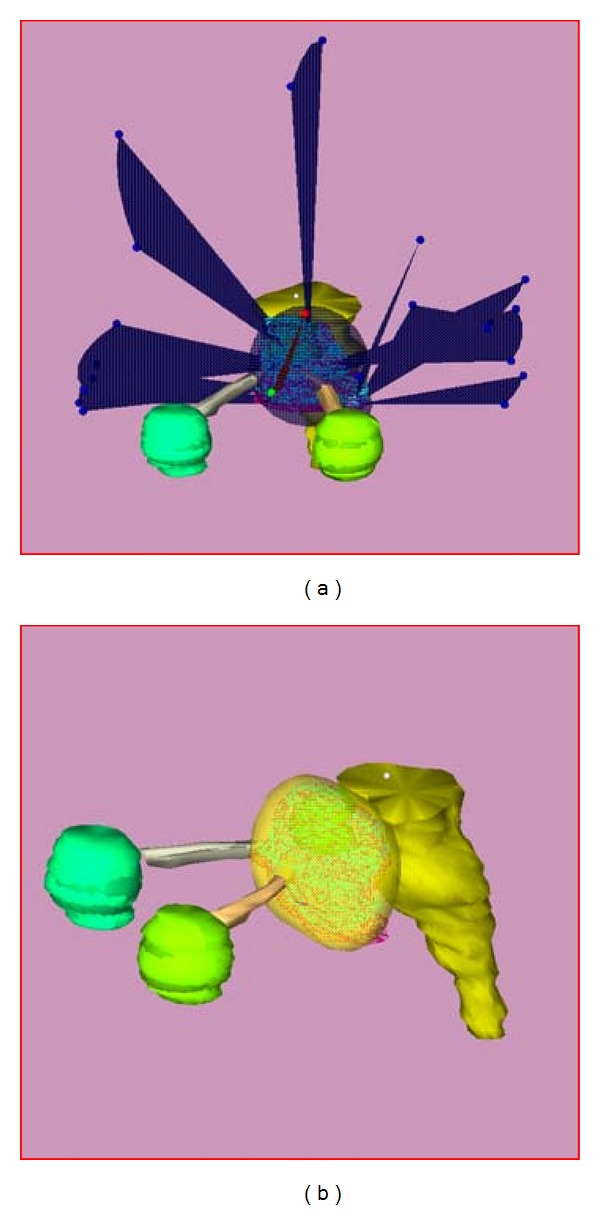
Treatment planning for SRT, using 3 isocenters. The right image shows isodose volume received 50 Gray in 25 fractions.

In 2009, he developed no light perception of the right eye. MRI brain revealed progression of tumor invading the optic nerve and right cavernous sinus. Resurgery with tumor decompression at the right optic nerve was done in August 2009. Pathological report was pituitary adenoma. After resurgery, he was referred to our hospital. Regarding to no any other treatment option for this patient, we decided to reirradiate the patient with stereotactic radiation therapy, conventional fractionated with the total dose of 50 Gray in 25 fractions to the tumor. The SRT technique used the linear accelerator base system (6 MV dedicated LINAC, Varian; with X-Knife planning system version 3 & 4, Radionics). The relocatable Gill-Thomas-Cosman frame was used for patient immobilization and target localization. Individual treatment planning was done based on a contrast-enhanced CT scan, 1.5 mm slice thickness, with gadolinium enhanced MRI. The treatment planning is presented in Figure 1. The patient could tolerate the treatment very well. No acute complication was observed.

**Figure 2:**
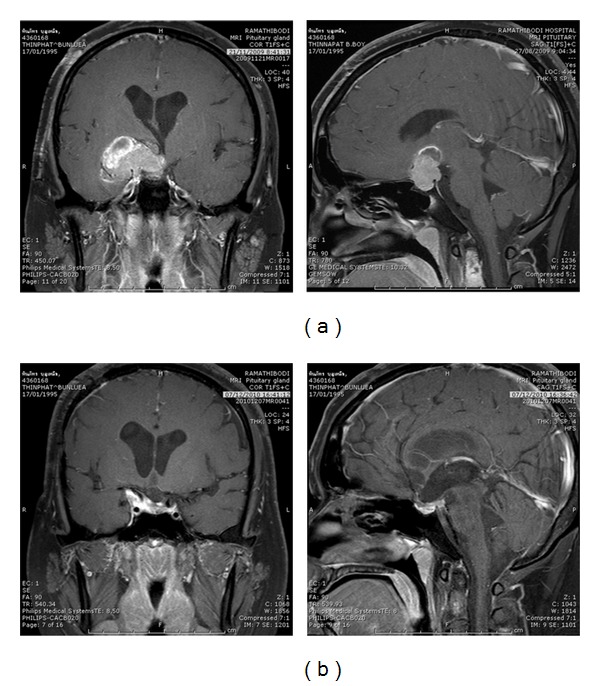
Gross tumor volume of a pituitary adenoma before SRT (a) and 3 months after SRT (b), representing a significant decreasing in size of tumor.

At 3 months after SRT, the tumor volume was reduced significantly on follow-up brain MRI as compared to pre-SRT imaging (Figure 2). 

## 3. Discussion

 Stereotactic radiation therapy can be delivered either by single fraction radiosurgery (SRS) or by fractionated stereotactic radiotherapy (SRT). If tumor closed to the optic apparatus, SRT should be used to avoid the risk of optic nerve injury from radiation. In most cases of nonfunctioning pituitary adenoma, the goal of radiation therapy is to arrest tumor growth. Partial shrinkage and less commonly complete resolution may occur in approximately two third of cases but need to be followed over a longer period of time after radiation effect becomes evident [4, 5]. However, in this case report, the rapid and significant tumor reduction was found at a shorter period of time about 3 months after radiation, which is unusual presentation from most of benign tumors. Typically, in a slow growing tumor such as pituitary adenoma, the effect of radiation may become evident over a longer period of time. A gradual reduction in tumor volume may be explained by radiobiological effects, considering radiation-induced DNA damage with cell reduction becoming apparent only after subsequent cellular division or programmed cell death. Another effect contributing to the observed tumor volume reduction over time might be due to chronic ischemic effects of radiation-induced vasculopathy. Kopp et al. [6] reported an inverse correlation between time after radiotherapy and relative tumor reduction similar to the finding by Tung et al. [7]. The mean reduction in tumor volume at 6 months after radiosurgery was 9% and increasing up to 50% at 4 years. One possible explanation for this rapid response might be related to a rapid growth of this tumor cells, resulting of rapid response of radiation effect.

Finally, we have assumed that some patients with nonfunctioning pituitary adenoma treated with radiation may show rapid and significant reduction in size of tumor. In routine practice, we did not perform MRI at early time point after radiotherapy and most of the patients were examined by MRI ranging from 28 to 72 months after radiotherapy. This paper might be used to support the early time point for MRI assessment such as 6 or 12 months after radiation, especially in case of rapid growth tumor or in cases of children.
