# Correction to “Rapid and Significant Reduction in Size of Pituitary Adenoma in Children Treated with Fractionated Stereotactic Radiation Therapy: A Case Report”

**DOI:** 10.1155/crie/9759156

**Published:** 2026-02-25

P. Puataweepong and M. Dhanachai, “Rapid and Significant Reduction in Size of Pituitary Adenoma in Children Treated with Fractionated Stereotactic Radiation Therapy: A Case Report,” *Case Reports in Endocrinology* 2011, no. 1 (2011): https://doi.org/10.1155/2011/187839.

Figure 2a,b have been replaced to remove the patient’s identifiable information. The overall content and conclusions remain unchanged.

Figure 2Gross tumor volume of a pituitary adenoma before SRT (a) and 3 months after SRT (b), representing a significant decreasing in size of tumor.

**Figure:**
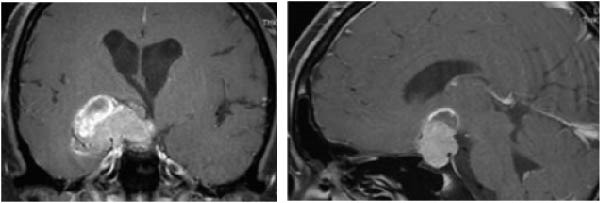
(a)

**Figure:**
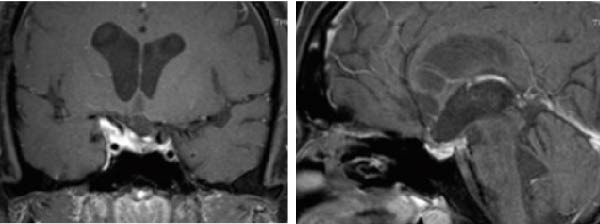
(b)

We apologize for this error.

